# X-ray Emission Hazards from Ultrashort Pulsed Laser Material Processing in an Industrial Setting

**DOI:** 10.3390/ma14237163

**Published:** 2021-11-24

**Authors:** Ulf Stolzenberg, Mayka Schmitt Rahner, Björn Pullner, Herbert Legall, Jörn Bonse, Michael Kluge, Andreas Ortner, Bernd Hoppe, Jörg Krüger

**Affiliations:** 1 Physikalisch-Technische Bundesanstalt (PTB), Department of Radiation Protection Dosimetry, Bundesallee 100, 38116 Braunschweig, Germany; mayka.schmitt-rahner@ptb.de (M.S.R.); bjoern.pullner@ptb.de (B.P.); 2 Bundesanstalt für Materialforschung und -prüfung (BAM), Materials Chemistry Department, Unter den Eichen 87, 12205 Berlin, Germany; kontakt-legall@t-online.de (H.L.); joern.bonse@bam.de (J.B.); joerg.krueger@bam.de (J.K.); 3 SCHOTT AG, Hattenbergstrasse 10, 55122 Mainz, Germany; michael.kluge@schott.com (M.K.); andreas.ortner@schott.com (A.O.); bernd.hoppe@schott.com (B.H.)

**Keywords:** X-ray emission hazards, ultrashort pulsed laser, radiation protection, industrial applications, protection housing, ambient dose rate, X-ray spectrum

## Abstract

Interactions between ultrashort laser pulses with intensities larger than 10^13^ W/cm^2^ and solids during material processing can lead to the emission of X-rays with photon energies above 5 keV, causing radiation hazards to operators. A framework for inspecting X-ray emission hazards during laser material processing has yet to be developed. One requirement for conducting radiation protection inspections is using a reference scenario, i.e., laser settings and process parameters that will lead to an almost constant and high level of X-ray emissions. To study the feasibility of setting up a reference scenario in practice, ambient dose rates and photon energies were measured using traceable measurement equipment in an industrial setting at SCHOTT AG. Ultrashort pulsed (USP) lasers with a maximum average power of 220 W provided the opportunity to measure X-ray emissions at laser peak intensities of up to 3.3 × 10^15^ W/cm^2^ at pulse durations of ~1 ps. The results indicate that increasing the laser peak intensity is insufficient to generate high dose rates. The investigations were affected by various constraints which prevented measuring high ambient dose rates. In this work, a list of issues which may be encountered when performing measurements at USP-laser machines in industrial settings is identified.

## 1. Introduction

The use of ultrashort laser pulses in air for material processing has many advantages such as the lateral and vertical precision of the surface contours down to the nanometer range and the high reproducibility of the laser-generated structures [1]. Due to the progressive development in the laser sector, average powers in the kW range with pulse repetition rates exceeding the MHz level are currently available [2]. Machining with high-intensity laser pulses can be accompanied by the generation of a near-surface electron plasma due to absorption and ionization of the material, a subsequent plasma heating by the laser pulse, and finally an interaction of “hot” plasma electrons with the processed material, leading to continuous and characteristic X-ray emissions.

The fact that ultrashort sub-ps pulsed laser material interaction can lead to X-ray radiation has been well established since the late 1980s [3,4,5,6,7,8]. Twenty years ago, X-ray emission was reported as an unwanted secondary effect during femtosecond laser micromachining of copper in air using kHz repetition rates [9,10]. The authors registered X-ray dose rates requiring radiation protection measures. The amount of this X-ray radiation is determined by the laser parameters (pulse duration, peak intensity, pulse energy, wavelength, and polarization), the workpiece (atomic number and surface preparation), and the laser process management (scanning or stationary regime, laser turning, etc.). The use of laser peak intensities above 10^13^ W/cm^2^ in combination with laser pulse repetition rates in the few 100 kHz range can already lead to X-ray dose rates clearly exceeding the permitted limits for members of the public. Tungsten and steel in particular show significant X-ray emission [11,12,13,14,15,16,17,18,19]. In [11], it was demonstrated that other materials such as aluminum and glass show significantly lower X-ray emissions. The measured dose rate for an aluminum target was approximately two orders of magnitude lower than the dose rate of steel and tungsten. For a fixed intensity, an increase in the measured dose rate with a raising atomic number *Z* was observed as a general trend. Under typical ultrashort pulse laser machining conditions in the intensity range of 10^13^ to 10^15^ W/cm^2^, collision-less resonance absorption appears to be the driving X-ray generation mechanism [17].

It has been shown very recently, experimentally, that significantly higher X-ray dose rates can be generated during USP-laser machining when using a laser burst mode instead of the single-pulse mode utilizing the same total laser intensity. The increase in X-ray yield was attributed to the interaction between the ultrafast laser radiation and an ablation cloud or high-density plasma [19,20]. This special regime was not investigated within the framework of this paper.

Due to the possible emission of ionizing radiation during material processing with a USP-laser machine, radiation monitoring and protection measures for staff working on such machines must be implemented. One such measure is dosimetry measurements outside of the protective housing or structural radiation protection features during the authorization process for operation of USP-laser machines. To ensure conservativity during the radiation protection inspection, the material processing scenario with maximum radiation exposure and the highest photon energies of laser-induced X-ray radiation would typically have to be employed. However, due to the large number of relevant laser and processing parameters, determining and setting up a worst-case scenario is time-consuming and impractical during regular safety inspections. Nevertheless, for consistent and reproducible inspection results, a reference condition must be defined. In the case of USP-laser machines, this is especially important as laser–plasma interactions and the subsequent energy spectra and intensities of the X-ray emission strongly depend on various operational parameters such as laser parameters, the manufacturing process and its geometry, and the previously mentioned laser-irradiated material.

The possible reference scenario used here is based on the laser surface processing of a metal sample such as steel or tungsten. For the measurements, a bidirectional configuration is used. During this process, grooves with a certain length (e.g., 15 mm) are machined into the target material. After finishing one groove, the material is moved by one focus diameter in the direction perpendicular to the groove, and another groove is manufactured in the opposite direction. This process is repeated until a surface scan area of, for example, 15 × 15 mm^2^ has been covered. This includes the ablation of a single “process layer”. To increase the duration of the laser processing, either the size of a single layer can be increased or multiple layers can be vertically stacked. However, as demonstrated in [13] processing multiple ablated layers can lead to changed X-ray emission characteristics due to the modified surface topography after the machining of each layer. Such a reference material processing scenario has the advantage that the direction of the radiation field is well known. Due to intrinsic absorption in the target material, the highest dose rates of the laser-induced radiation are measured in the opposite direction to the movement of the laser, i.e., parallel to the laser ablated grooves [11,13,14].

A reference scenario employed during radiation protection inspections should be conservative with respect to the ambient dose rate of the X-ray emission and the maximum photon energy of the X-ray spectrum. This ensures that, during a radiation protection evaluation of the protection housing of the USP-laser machine, the transmission of the ionizing radiation is not underestimated. According to the literature [11], one of the most important parameters for generating laser-induced X-ray radiation with large ambient dose rates is the laser peak intensity. Increasing the laser peak intensity via the laser pulse energy will lead to an increase in the ambient dose rate of the emitted laser-induced X-ray radiation. It has also been shown in previous studies that the emitted dose rate strongly depends on the specific material of the workpiece [11,14]. The highest dose rates have been observed when employing either steel or tungsten as targets. Therefore, it can be assumed that, during radiation protection inspections on a USP-laser machine, the best approach would be to use either one of these two materials and to employ the maximum available laser pulse energy at a given laser pulse duration.

Another important prerequisite of the reference scenario is a temporally stable X-ray emission with an almost constant dose rate level during the radiation protection inspection. Additionally, the geometry of the entire radiation field should be well known and have a large area of homogeneous X-ray emission. Both requirements ensure reliable radiation protection measurements over the whole area of the protection housing.

The requirements for evaluating radiation protection inspection approaches at USP-laser machines under realistic industrial settings were met at the company SCHOTT AG. A USP-laser of 1 ps pulse duration, a wavelength of 1030 nm, and an average power of 220 W embedded in an aluminum protection housing was available for the measurements. The theoretically achievable peak intensity of 3.3 × 10^15^ W/cm^2^ is comparatively high and normally used for the processing of transparent glasses making use of nonlinear absorption mechanisms. So far, only Schille et al. have employed higher laser peak intensities of up to 5.2 × 10^16^ W/cm^2^ in a recent study [19]. The aluminum protection housing at the USP-laser machine at SCHOTT AG was an interesting test subject as the protective effect is expected to be much smaller than for steel enclosures. Lastly, some interesting safety features, as explained later, are present in the machine.

Next, the experimental setup, some issues encountered during the measurements, and the results for the measurements in an industrial setting at SCHOTT AG with two different experimental setups are summarized. In particular, constraints imposed on the placement of the X-ray measurement equipment due to safety features inside the protection housing had a large impact on the measurements. Because of these issues and despite experience with the generation of laser-induced X-ray radiation, the dose rates measured at SCHOTT AG are lower than those obtained during measurements at similar laser peak intensities of other groups.

## 2. Materials and Methods

### 2.1. USP-Laser Machine

**Figure 1:**
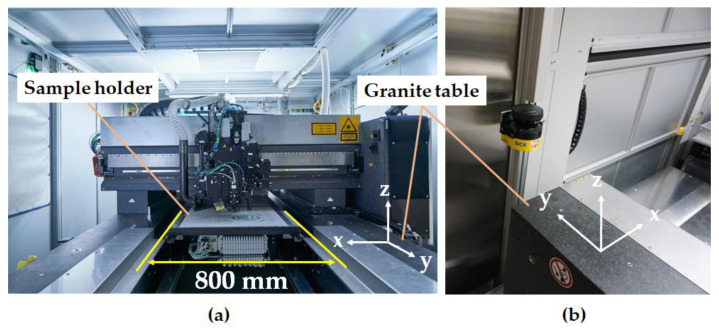
(**a**) Interior of the microSHAPE^TM^ USP laser system at SCHOTT AG. The system is accessible and equipped with several safety systems that are intended to prevent staff from entering the machine during laser operation. One of these safety systems is the Sick laser scanner device (**b**) installed in the wall in front of the operating machine system. The monitored area covers the region between 10 cm and 30 cm above the granite table.

The measurements were performed in a walk-in microSHAPE^TM^ machine (3D-Micromac, Chemnitz, Germany), which has an XHE 200 W (Amphos, Aachen, Germany) laser system. A photo of the interior of the system is shown in Figure 1.

The laser was used with fixed optics (no laser scanner was employed) that focused the laser beam, using standard processing laser parameters, onto the workpiece, creating a spot diameter of about 10 µm. The focus diameter was calculated by the control system of the USP-laser machine from manufacturer information of the laser beam diameter and the different focal lengths of lenses in the focusing optics system. Since the movement of the laser spot and the optics (*x*- and *z*-direction) on the workpiece had to be carried out in relation to the sample holder station (*y*-direction) by translating the sample, the feed speed rate had to be set considerably low at 100 mm/s. The sample holder has a Y-stage assembly and provides a focal plane with a working area of up to 0.5 m^2^ (see Figure 1a).

The industrial USP-laser machine used in this work was designed as a walk-in configuration; therefore, it has several safety mechanisms implemented to prevent the trespassing of people during laser operation. Figure 1b shows a laser safety scanner from Sick (Düsseldorf, Germany) [21] placed at the entrance of the microSHAPE^TM^ USP-laser machine. This device uses the time-of-flight principle (ToF) to monitor the room for objects and people and was particularly relevant for setting up the position of the X-ray measuring devices for this study.

### 2.2. Sample Workpiece and Processing Parameters

**Figure 2:**
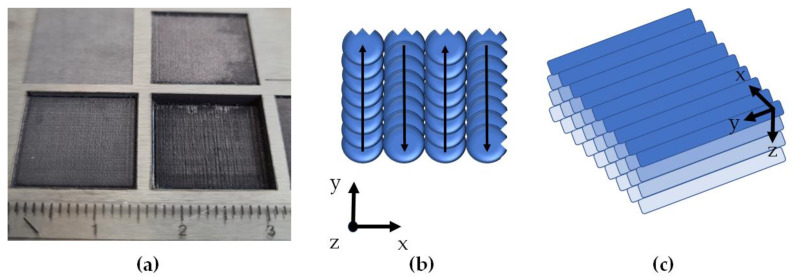
(**a**) The 15 × 15 mm^2^ scan areas after USP-laser processing with the microSHAPE^TM^ machine at peak intensities of 1 × 10^15^ W/cm^2^ and above (see Table 1). The visibly different depths of the processed areas are caused by different numbers of processing steps and variations of the laser pulse energy. The square in the top left corner was processed at PTB with a much smaller peak intensity of 3.0 × 10^14^ W/cm^2^. (**b**) Schematic of the 2D bidirectional laser spot movement with respect to the coordinate system as given in Figure 1. In (**c**), multiple processing layers in the *z*-direction are indicated.

As described above, a surface process on a tungsten plate was employed as a reference scenario. The machined areas were 15 × 15 mm^2^ squares (Figure 2a), processed with laser beam scanning in a bidirectional sequence (Figure 2b) *n* times on the tungsten plate (Figure 2c) without readjusting the distance between optics and sample surface during measurements. The laser pulse duration was 1 ps. Three different configurations of laser pulse energy and repetition rate were measured. These configurations are summarized together with the calculated peak intensity and laser spot distance (in *y*-direction) in Table 1. For comparison, the parameters of measurements taken with two other experimental setups using an HR50 laser (Coherent, Santa Clara, CA, USA) and a GL.evo (GFH GmbH, Deggendorf, Germany) are also included in Table 1. The offset between two adjacent lines corresponded to one focus diameter (in *x*-direction). With these settings, two adjacent processing points were approximately 0.250 µm to 2 µm apart along a line. Due to the large overlap between two adjacent machining spots, deep holes were created on the material surface, and a large amount of material was overall removed per laser scan.

In the first processes, to carefully test how the material behaves and not to damage the optics, only a fraction of the power was used. The processing areas produced by the microSHAPE^TM^ USP-laser machine were visibly deeper than those of the GL.evo USP-laser-machine (Figure 2a, top left) due to the high average laser output power of 220 W of microSHAPE^TM^ USP-laser machine, the large available peak intensities, and the large overlap between adjacent laser spots at a scan velocity of 100 mm/s. The GL.evo machine located at PTB uses a laser with a much smaller average power and peak intensity. For example, during one measurement with the microSHAPE^TM^ USP-laser machine, the overall removed material thickness of tungsten was approximately 500 µm (Figure 2a, bottom right) after four processing layer repetitions (*z*-direction in Figure 2c) in comparison to only a few µm after an equal number of repetitions at the GL.evo machine.

**Figure 3:**
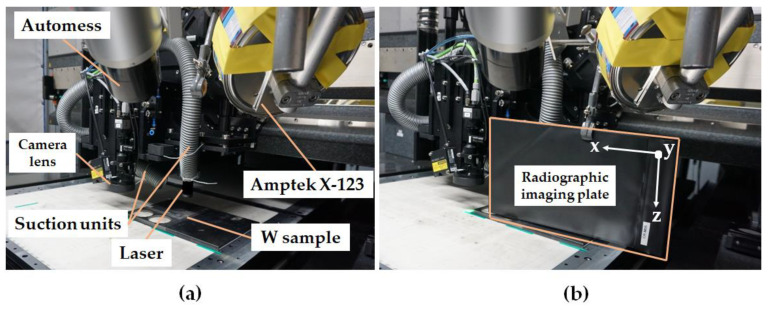
(**a**) Experimental setup and positioning of the measuring devices in the interior of the USP-laser machine. The Amptek X-123 spectrometer was placed in a tinplate box with an opening in front of the window and additionally covered with seven layers of 13 µm thick aluminum foil for shielding to prevent electromagnetic compatibility influences. (**b**) Placement of the radiographic imaging plate to detect the direction and homogeneity of the X-rays emission during USP-laser processing.

Since the removal rate in the reference scenario was much larger than those routinely applied by SCHOTT AG for glass processes using this machine, the exhaust system had to be temporarily reinforced during the measurements by bringing the suction opening closer to the processing position. This was realized with the help of an additional vacuum pipe and the adjustment of the machine’s suction system to bring it as close as possible to the plasma region. Nevertheless, after each processing step (with two to four ablation layer repetitions), the lens of the laser focusing optics had to be cleaned to prevent smoke traces that could reduce the focusing quality and laser energy delivered to the target. In Figure 3, one can see the experimental setup in detail. To be able to estimate the radiation direction and the size of the X-ray radiation field, a radiographic imaging plate from a computer radiography scanner was attached to the interior of the USP-laser system near to the processing point, as shown in Figure 3b.

### 2.3. Placement of the Measurement Equipment

As mentioned in Section 2.1, the existence of a coupled safety scanner monitoring system in the investigated microSHAPE^TM^ USP-laser machine made the placement of the measuring instruments challenging. The area monitored by the laser safety scanner encompassed a region with a height of about 10 cm to 30 cm above the granite table and reaching close to the laser head in *y*-direction of the machine (see Figure 1b). Thus, if any object is detected by the monitoring laser scanner in this region (from now on called the “safety monitored area”), either the USP-laser itself would not start, or it would immediately be switched off as an emergency case during operation. Due to these restrictions, the standard constructions of PTB, which normally include the usage of laboratory tripods or mobile lifting bases, could not be applied here. Instead, an aluminum beam was fixed to the top of the USP-laser machine creating a gantry to hold the X-ray spectrometer and the dosemeter in a way to avoid the safety monitored area of the Sick laser safety scanner. Furthermore, because of the safety monitored area and the shielding by optical components, both devices could only be set up in approximately 45° to 60° angles to the direction of the movement of the laser relative to the sample workpiece, as shown in Figure 3. The distance between the dosemeter and the focus point of the USP-laser was 475 mm, while the distance between the spectrometer and the focus point was 385 mm.

### 2.4. Instrumentation

Table 2 gives an overview of the instruments used in this study to quantify laser-induced X-ray emissions while operating the USP-laser machines.

For measuring the ambient dose rate during USP-laser processing, the dose rate meter 6150AD-b/E from Automess—Automation and Messtechnik GmbH (Ladenburg, Germany) [22] was used. The dosemeters employed were calibrated in reference X-ray fields at PTB. As the lowest point for the energy calibration, a radiation quality of the narrow ISO X-ray series N-15 [23] with a mean energy of approximately 12.4 keV was used [24].

**Figure 4:**
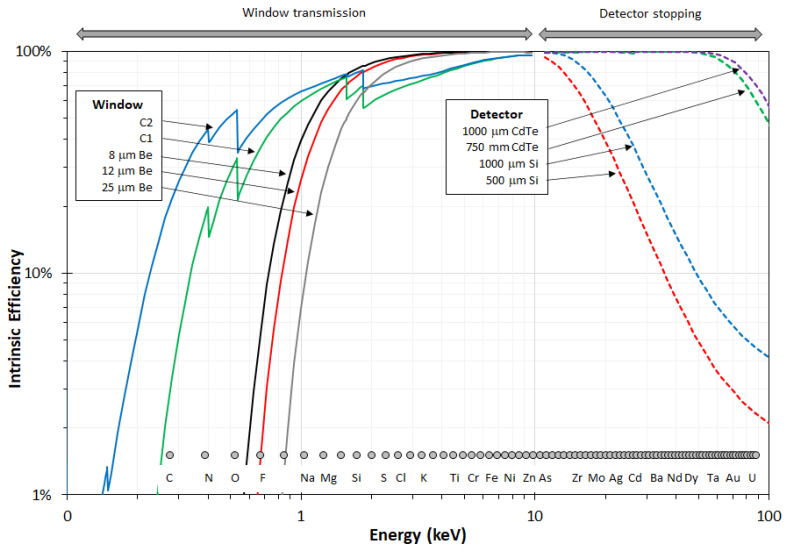
Comparison of the intrinsic efficiency of the X-123 detectors from Amptek. At low energies, the Be window limits sensitivity, and the 1 mm thick CdTe crystal (dashed violet curve) has a good efficiency up to 70 keV. © Amptek Inc 2021. Reprinted with permission.

For measuring the laser-induced X-ray energy spectrum using the tungsten plate as a workpiece, an X-123 CdTe spectrometer from Amptek Inc. (Bedford, MA, USA) [25] was employed. It was placed in a tinplate box and additionally covered with seven layers of 13 µm thick aluminum foil to assure electromagnetic compatibility (EMC) and prevent interference through unwanted electrical or electromagnetic effects in the readout electronics. The transmission as a function of photon energy for 90 µm of aluminum is shown later. The Amptek X-123 detector is based on a CdTe crystal with a thickness of 1 mm used as an X-ray detector [26]. The interaction probability (intrinsic efficiency) as a function of the photon energy for different Amptek detector systems is depicted in Figure 4. The dashed violet curve corresponds to the device used for the measurements at SCHOTT AG. As can be seen, it encompasses the energy region of interest for radiation protection on USP-laser machines up to 70 keV. For low energies, the efficiency is limited by a 100 µm Beryllium window, which is not included in Figure 4, and the additional 90 µm of aluminum in front of the device. Due to absorption in the window material and the aluminum, a slightly decreased efficiency of the detector can be expected for energies lower than 8 keV.

For monitoring the homogeneity and the location of higher concentrations of emitted X-rays during USP-laser machining, a 2D Radiography Scanner CR 35 NDT Plus system from DÜRR (Stuttgart, Germany) [27] was used. As shown in Figure 3b, the radiographic plate could be placed adjacent to the tungsten sample, around 10 cm away from the laser-induced plasma and just outside the safety monitored area.

## 3. Results

### 3.1. Monitoring the Generation of X-ray Emissions

**Figure 5:**
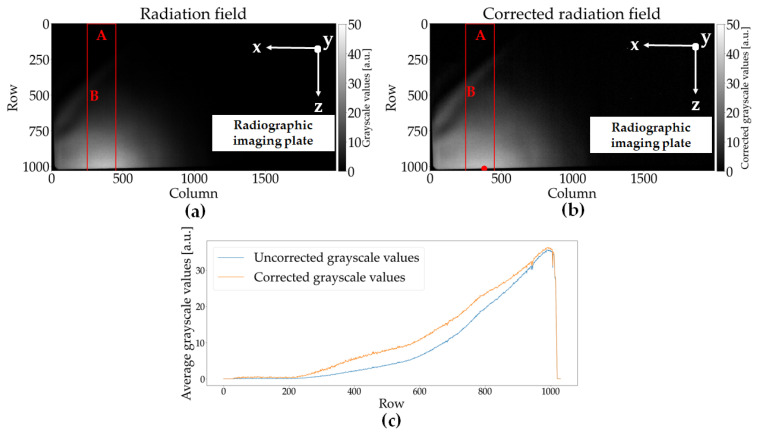
(**a**) Uncorrected and (**b**) distance-corrected grayscale image captured with the radiographic imaging plate. Both images show that a large part of the X-ray radiation is emitted very flat and almost exclusively parallel to the sample plate in the *y*-direction. The edge of the imaging plate can be seen very well as a sudden drop in the grayscale values in the lower and left part of the image. The red rectangles in (**a**,**b**) show the area of the pixels used for the profile curve plotted in (**c**). The red dot in (**b**) marks the position of the shortest distance to the plasma used for the calculation of the distance correction. (**c**) Cross-section of the grayscale values along the rows of the corrected (orange curve) and uncorrected (blue curve) grayscale images. The continuous decrease in grayscale value with a decreasing row number in the profiles indicates that a large part of the laser-induced X-rays is emitted in a very flat angle close to the horizontal plane.

The recorded grayscale image of the radiography image plate is depicted in Figure 5a. The area of each pixel of the grayscale image is approximately 200 × 200 µm^2^. As the radiographic imaging plate was located very close to the source of the laser-induced X-ray emission (see Figure 3b), the grayscale image pixels had to be corrected for different distances from the plasma (see Figure 5b).

The shortest distance from the plasma was 10 cm, and the position of this point of shortest distance on the imaging plate is marked with a red dot on the lower left side of the grayscale image in Figure 5b. As the grayscale values are proportional to the local intensity of the X-ray emission and the intensity of a point source decreases with the square of the distance from the source, the distance-corrected grayscale values can be calculated as follows:

*G(r*_0_*)* = *G*(*r*) *r*^2^/*r*_0_^2^, (1)

where *G*(*r*_0_) is the corrected grayscale value at the reference distance *r*_0_ (in this case, the shortest distance of 10 cm), and *G*(*r*) is the measured grayscale value at a distance *r*. The distance *r* of each grayscale value can be calculated from the row and column position (*u*, *v*) on the imaging plate, *r*_0_ and the row and column values (*u*_0_, *v*_0_) of the reference distance on the grayscale image (in this case (*u*_0_, *v*_0_) = (371, 1021)), and the pixel pitch *p*.

*r*^2^ = *r*_0_^2^ + *p*^2^ (*u* − *u*_0_)^2^ + *p*^2^ (*v* − *v*_0_)^2^. (2)

Further corrections due to additional absorption in air for larger distances from the plasma and a possible angle dependency of the radiographic imaging plate have not been applied. The area with the highest X-ray emissions, characterized by bright gray pixels, is located close to the lower edge of the imaging plate. Accordingly, the X-ray radiation is emitted in a very flat angle to the horizontal plane. In addition, as expected, the emission also appears to be largely parallel to the line of movement of the USP-laser (*y*-direction). The edge of the radiographic imaging plate is located in the lower and left part of the grayscale image. The different areas of the radiation field can also be seen in the comparison between the distance-corrected and uncorrected *z*-profile (the image pixels employed to generate the profile are indicated by the red rectangle in Figure 5a,b) of the grayscale values depicted in Figure 5c. From the measurements performed with the radiography imaging plate, one can conclude that the placement of the dosemeter and the spectrometer devices was not optimal regarding the maximum emission of the X-ray radiation, i.e., they were not directly installed in the maximum region but at the edge of the radiation field. However, as discussed in Section 2.3, this was the best arrangement possible for this USP-laser machine.

### 3.2. Obtained Energy Spectra and Dose Rates

**Figure 6:**
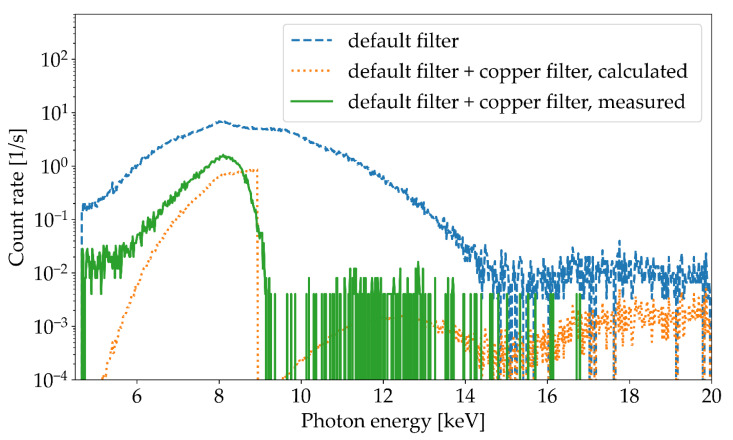
Pulse height spectra measured with the Amptek X-123 CdTe spectrometer during tungsten flat surface processing using a laser pulse energy of 550 µJ, a repetition rate of 400 kHz, a pulse duration of 1 ps, and a focus diameter of 10 µm. Shown are the measured spectra without any additional copper filter (blue curve) and with a copper filter (green curve), as well as the calculated spectrum based on the convolution of the spectrum without copper filter with the transmission function of 50 µm copper (orange curve).

Figure 6 depicts pulse height spectra from laser-induced X-ray emissions with and without a 50 µm thick copper filter in addition to the default aluminum filter with an overall thickness of approximately 90 µm. The spectra were measured with a laser pulse energy of 550 µJ, a pulse duration of 1 ps, and a repetition rate of 400 kHz. The mean X-ray photon energy values obtained during the USP-laser processing with and without a 50 µm thick copper foil were approximately 8.1 keV and 8.9 keV, respectively.

Moreover, for comparison, the expected spectrum after transition of a 50 µm thick copper filter was calculated. This calculated spectrum (orange curve in Figure 6) is based on the measured spectrum without a copper filter (blue curve) multiplied by a transmission function *T*(*E*, *d*) in each energy channel. The function *T*(*E*, *d*) describes the exponential attenuation of photons traversing a material [28] and is given by

**Figure 7:**
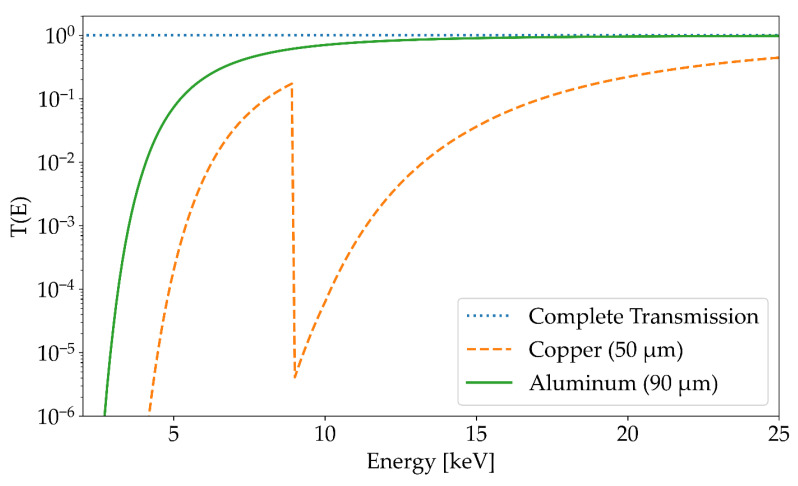
Transmission function *T*(*E*, *d*) (see Equation (1)) for 50 µm of copper (orange dotted curve) and 90 µm of aluminum (solid green curve) as a function of the energy. For the calculation, the list of mass attenuation coefficients from the National Institute of Standards and Technology was used. The values were logarithmically interpolated to get a value for each energy channel of the X-123 spectrometer. The rapid decrease at approximately 9 keV is caused by the sudden increase in the attenuation coefficient of copper due to the Cu-K edge.

*T* (*E*, *d*) = e^−(*µ* (*E*)/*ρ*) × *ρd*^, (3)

where *d* and *ρ* are the thickness and density of the material (in this case 50 µm of copper foil), respectively, and *µ(E)/ρ* is the energy-dependent mass attenuation coefficient of the material. For the calculation, the list of mass attenuation coefficients from the National Institute of Standards and Technology was used [28]. These tabulated values were logarithmically interpolated to obtain a corresponding value of the mass attenuation coefficient for each energy channel of the Amptek X-123 spectrometer. The *T*(*E*, *d*) for 50 µm of copper and 90 µm of aluminum as a function of the photon energy, together with the curve for no attenuation, i.e., *T*(*E*, *d*) = 1, is depicted in Figure 7.

As seen in Figure 6, the pulse height spectrum determined using the transmission function agrees very well with the pulse height spectrum measured with a 50 µm thick copper filter. The sharp drop in the spectrum at around 9 keV, which is caused by the absorption edge of the K-shell of copper, is clearly visible. The deviation for higher energies exceeding 15 keV is most likely caused by background noise and the pileup effect. This is also supported by the fact that the pulse height spectrum with an additional copper filter has no entries in this energy range. The pileup effect arises if multiple X-ray photons hit the single photon detector in the timeframe of the processing time and are registered as one single photon with correspondingly higher energy; this can be minimized by positioning the spectrometers at a large distance to the X-ray radiation source. As verified by the good agreement between the calculated and measured curves in Figure 6, further X-ray emission spectra were measured without the copper filter in front of the spectrometer window.

**Figure 8:**
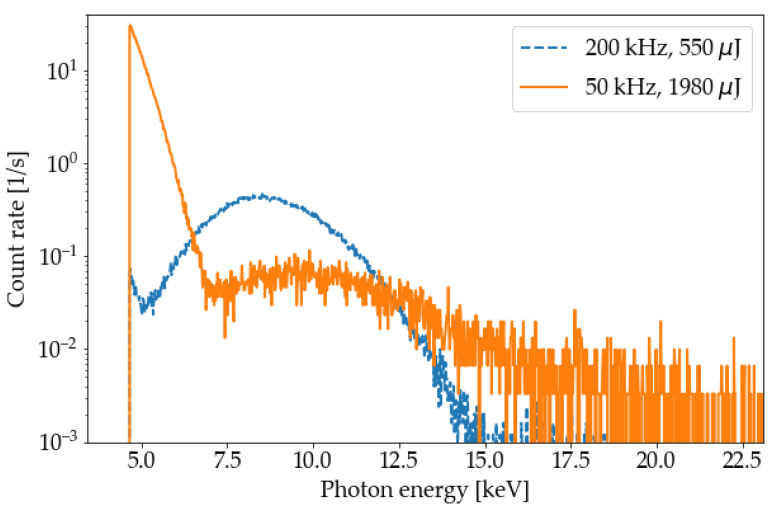
Pulse height spectra measured for the combination of two different USP-laser pulse energies and laser repetition rates: 550 µJ and 200 kHz (blue curve), 1980 µJ and 50 kHz (orange curve). The number of photons per channel with an energy bandwidth of 20 eV was divided by the total USP-laser processing time, to make the two spectra comparable. The increase in irradiance due to the higher pulse energy causes an energetic shift of the maxima of the spectra to higher photon energies. The increase of count rate in the low-energy region (between 5 keV and 7 keV) for the 50 kHz process is most likely caused by electronic noise due to an insufficient shielding of the CdTe detector. Overall, the recorded number of X-ray photons per second is very low, which was also confirmed in separate dose rate measurements with the Automess 6150AD-b/E dosemeter (see Table 1).

Figure 8 shows the comparison of the X-ray emission spectra for a fixed average laser power but two different combinations of pulse energy and repetition rate configured in the microSHAPE^TM^ USP-laser machine. The first measurement (orange curve) took place at a laser repetition rate of 50 kHz and a laser pulse energy of 1980 µJ, while the second measurement (blue curve) was carried out at a laser repetition rate of 200 kHz and a laser pulse energy of 550 µJ. The respective laser peak intensities of the laser pulses were 3.3 × 10^15^ W/cm^2^ and 0.9 × 10^15^ W/cm^2^. To be able to compare the measurements as plotted in Figure 8, the number of photons (counts) per channel with an energy bandwidth of 20 eV was divided by the accumulation time in seconds of each experiment.

An increase in the laser pulse energy and the associated increase in the peak intensity of the laser pulses led to a shift of the spectrum to higher X-ray photon energies, as shown by the two combined spectral measurements in Figure 8. Accordingly, the average power of the laser, which was nearly identical for both configurations, cannot be employed as a simple scaling parameter for the emitted X-ray radiation as the photon energy spectrum is affected by the variation of the repetition rate and the pulse energy. The increase in counts in the low-energy region (between 5 keV and 7 keV) was most likely a consequence of an insufficient shielding of the CdTe detector and may have been caused by the pulsed intense electromagnetic laser plasma field. However, X-ray emissions at such low energies (here below 8 keV) would play only a minor role in relation to the hazard potential in enclosed USP-laser systems, because a large part of the photons would be absorbed in the ambient air and when passing through the protective housing and, thus, would not reach the operating personnel working on the machine.

Overall, the accumulated X-ray photon energy in the spectra was very low. This was also confirmed by the complementary dose rate measurements with the calibrated Automess 6150 dosemeter. The maximum measured ambient dose rates for the three different configurations at the microSHAPE^TM^ USP-laser system are listed in Table 1. During the surface process with a pulse energy of 1980 µJ, an ambient dose equivalent rate dH*(10)/d*t* of around 1 µSv/h was measured. These X-ray emission dose rates are much lower compared to the mSv/h reported recently in the literature for the same laser pulse duration and a lower laser pulse energy [11,12,18,19].

Further measurements were likewise carried out with the tungsten plate and a borosilicate glass sample, using a second experimental setup with a Coherent HR50 USP laser (Santa Clara, CA, USA). The Coherent HR50 laser has an average power of 35 W, a repetition rate of 200 kHz, and an emission wavelength of 1064 nm. For the radiation protection experiments, a pulse energy of 175 μJ and a laser pulse duration of 10 ps were applied; the focus diameter was approximately 20 µm, resulting in a peak intensity of approximately 1.0 × 10^13^ W/cm^2^. Thus, the peak intensity of the laser is significantly lower than that of the microSHAPE^TM^ USP-laser system with an output power of 220 W, reaching up to 3.3 × 10^15^ W/cm^2^.

**Figure 9:**
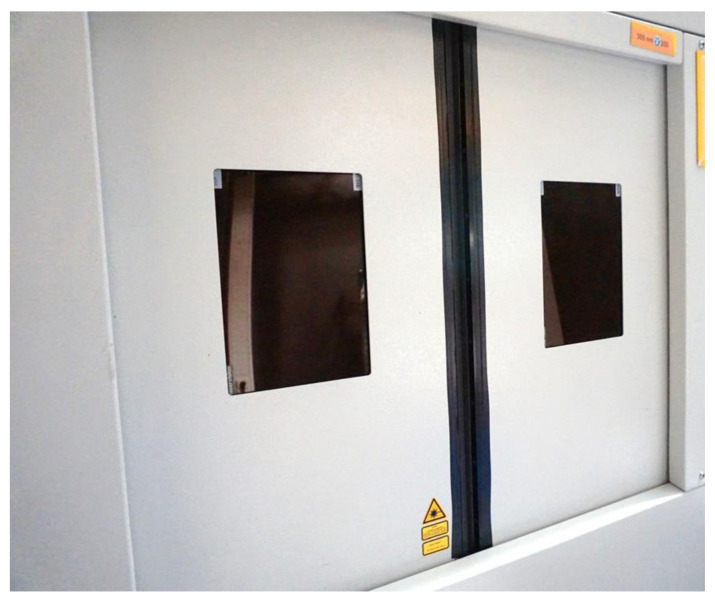
Photograph of the protection housing doors of the second experimental setup. The laser used in the system is a Coherent HR50 USP-laser with an average power of 35 W. The housing is made of 2 mm thick steel, and the windows are made of PMMA covered with a thin sheet of aluminum. Between the two doors there is a narrow area where, in the closed position, only rubber material lips join without any additional steel or aluminum layers.

The second setup with the Coherent laser was selected for further measurements because of the distinctive features of the 2 mm steel protection housing. It has an opening station comprising two doors with windows made of PMMA polymer covered by a thin aluminum layer (see Figure 9). Additionally, in a small slit region where the two doors join when closed, rubber material without any additional steel or aluminum layers is located. In this region, transmission of X-ray radiation is much less suppressed when compared to the surrounding steel protection housing.

On one hand, those are weak points in the protection housing that must be considered with regard to the transmission of X-rays. On the other hand, during the measurements with tungsten and glass targets, no deviation from the natural background radiation could be observed with the Automess 6150 dosemeter at a distance of 190 mm from the focus point of the laser. Measurements with the radiographic imaging plates and the X-123 CdTe spectrometer also showed no signal from laser-induced X-ray emissions.

## 4. Discussion

During measurements at the second experimental setup with a Coherent HR 50 laser, tungsten and borosilicate glass target material, and a peak intensity of approximately 10^13^ W/cm^2^, no emission of X-ray radiation could be observed with either the Automess 6150 dosemeter, the X-123 spectrometer or radiographic imaging plates at a distance of approximately 19 cm from the plasma. This is not unexpected as the generation of X-rays with energies large enough to traverse short distances of air is expected to be very inefficient for laser peak intensities of 10^13^ W/cm^2^ [11].

With the microSHAPE^TM^ USP-laser machine employing a tungsten workpiece, the measured dose rates are much smaller than expected for a laser-induced plasma generated at peak intensities of 3.3 × 10^15^ W/cm^2^ with tungsten as a target material. In the literature, typically, dose rates in the range of mSv/h are measured for such high peak intensities [18,19]. As can be seen from Table 1, the measured dose rate decreased with increasing pulse energy, which is unexpected. There are several possible explanations for these results.

First, the measurement equipment was installed in a nonoptimal position. As explained earlier, the main reason for this was a safety system, which monitored the interior of the USP-laser machine and prevented the installation of objects in a large area directly in front of the laser head and the focus point on the workpiece. As can be seen from the distance-corrected grayscale image (Figure 5b), the radiation field of the laser-induced X-rays is located near the horizontal plane and in the direction of the *y*-axis parallel to the lines. Due to the space limitations, the dosemeter and spectrometer had to be installed at a large angle of approximately 45° from the horizontal plane and approximately 45° to 60° to the processing grooves in the *y*-direction. Consequently, the radiometric measurement equipment was located at the fringes of the radiation field, where the X-ray dose rate is expected to be much lower than in the maximum of the field.

Additionally, the large rate of material removal caused large amounts of debris in the air near the workpiece and focusing optics. The exhaust system was not powerful enough to remove all remnants of the laser ablated material. Thus, some residual material was deposited on the focusing optics, degrading the quality of the laser spot and accordingly decreasing the energy deposited in the workpiece. Additional thermal effects which affect the focusing qualities of lenses of the focusing optics may also have had an impact. This degrading effect is most likely even worse when increasing the pulse energy and, therefore, increasing the amount of material removed per laser pulse. This would at least partially explain the low dose rate measurements at a pulse energy of 1980 µJ when compared to the measurements at 550 µJ.

Moreover, the scanning speed of the laser spot on the workpiece was comparably low with 100 mm/s. Other experiments in the literature used a much higher scanning speed ranging from 400 mm/s [19] to 1000 mm/s [11] to prevent large overlap of adjacent laser spots and, thus, the formation of very deep ablated groove structures. For the measurements at SCHOTT AG, the combination of a large laser pulse energy of 550 µJ and 1980 µJ and the slow movement speed of the laser spot led to the formation of very deep ablated groove structures. During the laser material processing, the plasmas at the bottom of these structures were deep within the material; therefore, most of the emitted low-energy X-ray photons were absorbed by the surrounding material. This effect is especially important here because of the placement of the measurement equipment due to safety restrictions. As can be seen from Figure 8, the difference between the high energy tails of the spectrum with pulse energies of 550 µJ and 1980 µJ is in the range of only a few keV. Accordingly, on one hand, increasing the pulse energy will only have a minor effect on the transmission of X-ray photons through the workpiece material, i.e., tungsten. On the other hand, increasing the pulse energy will lead to deeper groove structures and will, therefore, increase the amount of material that has to be traversed by the X-ray photons to reach the measurement equipment, resulting in an increased rate of absorption. This effect might explain the measurement of a lower dose rate at a higher pulse energy.

The acquired X-ray spectra from the highest investigated USP-laser pulse (1980 µJ) showed energies of up to 25 keV, which is consistent with other measurements at lower and similar peak intensities [11,14]. The measured spectra were tested by applying an additional copper filter with a thickness of 50 µm. In a comparison between the measured spectrum with the copper filter and the expected spectrum calculated from the spectrum and the transition function of 50 µm copper, a good agreement could be observed. Additionally, spectra of two different combinations of laser pulse energy and repetition rate were compared. As expected, a higher pulse energy and, consequently, a higher peak intensity led to a spectrum with higher maximum photon energies [14,29,30]. These measurements with nearly identical average laser powers but different pulse energies and repetition rates confirm that the average laser power cannot be employed as a simple scaling factor for the expected photon energy of laser-induced radiation.

The distribution of the distance corrected radiation field depicted in Figure 5b shows a clear maximum in the direction parallel to the laser movement along the processed lines in the material (*y*-direction). This result is in good agreement with previous measurements [11,13,14]. However, as can be seen from Figure 5c, the distribution of the X-ray emission has a maximum very close to the horizontal plane. In previous measurements, the maximum of the radiation field for the processing of tungsten was found to be at a larger angle of approximately 30° [11]. Two possible explanations for this deviation are presented here. First, this unexpected angular distribution of the radiation field might be caused by differences in laser peak intensity and, consequently, the large amount of residual material in the air near the plasma. This might, in turn, affect the angular characteristics of the plasma emission, due to absorption of the low energy X-rays in the material cloud. Another possibility is that, because of the very deep groove structures and the large overlap between adjacent laser spots, the plasma is located on the side wall of the groove and not at the bottom of the groove parallel to the surface of the target material. This would lead to a different alignment of the plasma density gradient, which significantly affects the emission characteristic of laser-induced X-rays.

As demonstrated, a fast, reliable, and reproducible investigation of the protection housing of the machine or structural radiation protection features may not be feasible using laser-induced X-rays. Consequently, other inspection concepts for future radiation protection tests of USP-laser machines must be considered. One alternative concept could be based on using a stable and continuous X-ray source such as an X-ray tube as a substitute for the laser-induced radiation. Requirements for this approach would be that the X-ray tube has a similar energy spectrum to the laser-induced X-ray emission. By varying the high voltage and current of the X-ray tube, the ambient dose rate and energy spectrum of the radiation can be determined, for which the transition radiation through the protection housing falls below legal dose rate limits. Such a mimicking of the laser-induced X-ray generation by conventional X-ray technology and already well-established inspection procedures appears feasible and would significantly facilitate the legal approval process of USP-laser processing machines by the local authorities.

## 5. Conclusions

The results and issues while performing dosimetric and spectrometric measurements of laser-induced X-ray emissions in a realistic industrial working environment clearly demonstrate that setting up a typical USP-laser machine for radiation protection inspections under reference conditions with worst-case X-ray emissions is very complicated and time-consuming. Optimizing the laser processing parameters, such as pulse energy and focus diameter with respect to the emission of laser-induced X-ray radiation, may lead to damage in the focusing optics or in other crucial components of the machine. Installed safety systems may prevent the person responsible for the radiation protection inspection from measuring under optimal conditions. Redeposition of ablated material on the optics of the laser processing system can additionally affect the measurements if the exhaust system is not powerful enough to remove unusually large amounts of ablated material produced during processing scenarios with high pulse energies and repetition rates.

All the adaptations and difficulties found during the measurement make it clear that, despite extensive experience with the generation of X-rays on USP-laser systems, it is not trivial to find a machining process on a third-party machine which can be employed as a reference scenario with high emissions of X-rays (or even the worst-case scenario). However, considering the numerous developments of USP-laser machines in industrial applications during the last few years and the number of USP-laser machines already in industrial use, the development of a reliable and reproducible radiation protection inspection of USP-laser machines is urgently required.

An alternative inspection concept for USP-laser machines could be based on using a stable and continuous X-ray source such as an X-ray tube as a substitute for the laser-induced radiation during the investigation of the machine housing.

## Figures and Tables

**Table 1:** Summary of laser processing parameters and corresponding measured dose rates for different USP-laser machines and their respective studied configurations. Pulse durations were 1 ps and 0.274 ps, and laser focus spot diameters were 12 µm and 33 µm (both manufacturer information) for the setups operated at SCHOTT AG and at PTB, respectively. The wavelengths of all lasers listed here were approximately 1 µm. The maximum ambient dose rate measurements at SCHOTT AG were conducted at a distance of 475 mm from the laser processing location. The maximum ambient dose rate at the GL.evo machine of approximately 2.1 mSv/h was measured at a distance of 160 mm during a laser turning process. The uncertainties listed for the dose rate values were calculated via error propagation of the calibration factor of the dosemeter.

USP-Laser Machine		Repetition Rate (kHz)	Pulse Energy (μJ)	Distance of Laser Spots (µm)	Peak Intensity (W/cm^2^)	Max. dH*(10)/d*t* (µSv/h)
Model	Operator					
3D-Micromac microSHAPE^TM^	SCHOTT AG	400	550	0.25	9.1 × 10^14^	128 ± 8
		200	550	0.5	9.1 × 10^14^	24 ± 2
		50	1980	2	3.3 × 10^15^	0.90 ± 0.06
Second setup, Coherent HR50 laser	SCHOTT AG	200	175	5	1.0 × 10^13^	Natural background radiation
GFH GmbH GL.evo	PTB	50	371	10	3.0 × 10^14^	Up to 2.1 × 10^3^

**Table 2:** Overview and description of the measurement instruments. The optimal energy range of the Automess 6150 dosemeter lies above 20 keV. However, it is calibrated in an ISO radiation quality N-15 reference field at PTB.

Instrument	Dosemeter	Spectrometer	Radiography Scanner
Model	Automess 6150AD-b/E	Amptek X-123 CdTe	DÜRR CR 35 NDT
Optimal energy range	20 keV to 7 MeV	8 keV to 70 keV	Not specified
Measured property	Ambient dose rate dH*(10)/d*t*	X-ray pulse height spectrum	X-ray intensity

## Data Availability

The data presented in this study are available on request from the corresponding author.
